# Supplementary material for: COZOID: contact zone identifier for visual analysis of protein-protein interactions
Source: BMC Bioinformatics. 2018 Apr 6;19:125. doi: 10.1186/s12859-018-2113-6 (PMC5889581; doi:10.1186/s12859-018-2113-6)
Supplement: Supplementary file 2 — Software build. Executable binary file of the software tool. (ZIP 118,784 kb) [file 12859_2018_2113_MOESM2_ESM.zip › cozoid/platform/docs/testng-6.5.1-javadoc.zip!/org/testng/xml/XmlMethodSelector.html]

XmlMethodSelector (TestNG)


---


|  |  |  |
| --- | --- | --- |
| **Overview** \| **Package** \| **Class** \| **Tree** \| **Deprecated** \| **Index** \| **Help** | |  |
| **PREV CLASS**   **NEXT CLASS** | **FRAMES**    **NO FRAMES**     **All Classes** |  |
| SUMMARY: NESTED \| FIELD \| CONSTR \| METHOD | DETAIL: FIELD \| CONSTR \| METHOD |  |


---


## org.testng.xml Class XmlMethodSelector

```
java.lang.Object
  org.testng.xml.XmlMethodSelector
```

---

``` public class XmlMethodSelector extends java.lang.Object ```

This class describes the tag  in testng.xml.
Created on Sep 26, 2005

**Author:**
:   cbeust, Alexandru Popescu

---

**Constructor Summary**

`XmlMethodSelector()`


| **Method Summary** | |
| --- | --- |
| `boolean` | `equals(java.lang.Object obj)` |
| `java.lang.String` | `getClassName()` |
| `java.lang.String` | `getExpression()` |
| `java.lang.String` | `getLanguage()` |
| `int` | `getPriority()` |
| `int` | `hashCode()` |
| `void` | `setClassName(java.lang.String name)` |
| `void` | `setExpression(java.lang.String expression)` |
| `void` | `setLanguage(java.lang.String language)` |
| `void` | `setName(java.lang.String name)` |
| `void` | `setPriority(int priority)` |
| `java.lang.String` | `toXml(java.lang.String indent)` |

**Methods inherited from class java.lang.Object**

`clone, finalize, getClass, notify, notifyAll, toString, wait, wait, wait`

**Constructor Detail**

### XmlMethodSelector

```
public XmlMethodSelector()
```


**Method Detail**

### getClassName

```
public java.lang.String getClassName()
```

---


### setClassName

```
public void setClassName(java.lang.String name)
```

---


### setName

```
public void setName(java.lang.String name)
```

---


### getExpression

```
public java.lang.String getExpression()
```

:   **Returns:**: Returns the expression.

---


### setExpression

```
public void setExpression(java.lang.String expression)
```

:   **Parameters:**: `expression` - The expression to set.

---


### getLanguage

```
public java.lang.String getLanguage()
```

:   **Returns:**: Returns the language.

---


### setLanguage

```
public void setLanguage(java.lang.String language)
```

:   **Parameters:**: `language` - The language to set.

---


### getPriority

```
public int getPriority()
```

---


### setPriority

```
public void setPriority(int priority)
```

---


### toXml

```
public java.lang.String toXml(java.lang.String indent)
```

---


### hashCode

```
public int hashCode()
```

:   **Overrides:**: `hashCode` in class `java.lang.Object`

---


### equals

```
public boolean equals(java.lang.Object obj)
```

:   **Overrides:**: `equals` in class `java.lang.Object`


---


|  |  |  |
| --- | --- | --- |
| **Overview** \| **Package** \| **Class** \| **Tree** \| **Deprecated** \| **Index** \| **Help** | |  |
| **PREV CLASS**   **NEXT CLASS** | **FRAMES**    **NO FRAMES**     **All Classes** |  |
| SUMMARY: NESTED \| FIELD \| CONSTR \| METHOD | DETAIL: FIELD \| CONSTR \| METHOD |  |


---
